# Nitrogen-Doped Carbon Dots Alleviate Pesticide Toxicity in Tomato by Regulating Antioxidant Systems

**DOI:** 10.3390/ijms26209916

**Published:** 2025-10-12

**Authors:** Xu Zhang, Yu Xin, Hao Wang, Yuting Dang, Wenhui Wang, Yi Gao, Yu Han, Rongrui Kang, Qinghua Shi, Han Du

**Affiliations:** 1 State Key Laboratory of Crop Biology, College of Horticulture Science and Engineering, Shandong Agricultural University, Taian 271018, China; 2 College of Food Science and Engineering, Shandong Agricultural University, Taian 271018, China

**Keywords:** tomato, pesticides, carbon dots, xenobiotic detoxification, antioxidants

## Abstract

The overuse of pesticides has raised serious food-safety and environmental concerns. Carbon dots (CDs) can act as biostimulants by enhancing photosynthesis, thereby promoting plant growth and stress tolerance. However, their roles in plant pesticide detoxification remain unclear. This study synthesized nitrogen-doped carbon dots (N-CDs) with strong blue fluorescence, excellent biocompatibility, and no cytotoxicity observed in HEK 293T cells. The N-CDs were synthesized from 1.025 g citric acid and 0.379 g urea, producing particles with a size of around 2.42 nm and abundant hydrophilic groups. When applied to tomato plants, N-CDs (especially at 150 mg·L^−1^) significantly reduced chlorothalonil (CHT) residues affecting tomato, by up to 66%. Importantly, N-CDs also improved tomato plant growth, reversing the negative effects of CHT on key parameters such as height, leaf area, and biomass. Indeed, under CHT conditions, N-CDs significantly reduced the contents of malondialdehyde, superoxide, and hydrogen peroxide. In contrast, N-CDs significantly increased the activities of superoxide dismutase, peroxidases, catalase, and ascorbate peroxidase to 117.57%, 158.53%, 162.79%, and 152.23%, respectively. Notably, N-CDs dramatically changed the glutathione pool for tomato detoxification. Overall, this study synthesized the non-cytotoxic N-CDs that not only promote tomato growth but also alleviate CHT toxicity by strengthening the tomato’s antioxidant defense system.

## 1. Introduction

Pesticides are indispensable in modern agriculture for protecting crops against pests and diseases, thereby preventing substantial yield losses [1]. However, their frequent and excessive application has raised serious concerns regarding food safety, environmental contamination, and plant health [2]. A significant proportion of applied pesticides fails to reach the intended target pests and instead accumulates in ecosystems or enters the food chain. This issue is particularly acute in greenhouse cultivation, where limited exposure to UV light, rainfall, and airflow slows and impedes the natural degradation of pesticides, resulting in their persistence within edible plant tissues [3]. Conventional remediation methods such as washing and soaking are often ineffective for removing these internalized residues [3]. Therefore, minimizing pesticide residues has become an urgent priority that necessitates in-depth scientific study.

Pesticides, while essential for crop protection, can cause significant phytotoxicity in plants, including shoot tip atrophy, leaf necrosis, and growth inhibition. A key consequence of pesticide exposure is the excessive production of reactive oxygen species (ROS), such as superoxide anions (O_2_^•−^) and hydrogen peroxide (H_2_O_2_) [4]. Excessive ROS disrupt photosynthesis and electron transport, leading to oxidative damage to lipids, proteins, and nucleic acids [5]. To mitigate these toxic effects, plants have evolved complex detoxification and antioxidant defense systems [6]. The detoxification of pesticides in plants generally proceeds through three coordinated phases. In Phase I, xenobiotics are modified by enzymes such as cytochrome P450s and peroxidases (POD) [7]. Phase II involves conjugation of the activated compounds with reduced glutathione (GSH) or sugars via enzymes like glutathione S-transferase (GST) and glycosyltransferase (GT), which reduces toxicity and enhances solubility [8]. In Phase III, the conjugated products are transported into vacuoles or the apoplast by ABC transporters for sequestration and degradation [9]. Parallel to this metabolic detoxification, ROS are scavenged by enzymatic antioxidants such as superoxide dismutase (SOD), catalase (CAT), POD, and ascorbate peroxidase (APX) [10]. Among these antioxidants, GSH plays a central role in both detoxification and maintaining redox homeostasis and cellular signaling [10]. It exists in both reduced GSH and oxidized GSH disulfide (GSSG) forms, with the GSH/GSSG ratio serving as a key indicator of oxidative stress [11]. GSH conjugates with electrophilic pesticide intermediates via its thiol group in a reaction catalyzed by GST [12]. The regeneration of GSH from GSSG is catalyzed by GSH reductase (GR), maintaining a functional detoxification cycle.

Although these intrinsic mechanisms provide plants with critical defense, they are often insufficient to fully counteract the damage caused by intensive pesticide use and ROS overaccumulation. This has driven the search for external strategies to support plant detoxification and stress management. Recent advancements in nanotechnology have revealed the potential of nanomaterials to promote plant growth and enhance stress tolerance [13]. Among them, carbon-based nanomaterials—particularly, carbon dots (CDs)—are emerging as promising candidates due to their high biocompatibility, water solubility, and environmental safety [14]. Although metal and metal oxide nanoparticles have been extensively studied, organic nanomaterials such as CDs remain relatively underexplored in agriculture applications. CDs, as zero-dimensional carbon nanomaterials, possess excellent photostability and a large surface area, making them suitable for diverse applications such as imaging, catalysis, and molecular delivery. Their role in agriculture, however, is only beginning to be explored. Emerging studies have shown that CDs can promote plant development by enhancing chlorophyll accumulation, improving root and leaf growth, and increasing the synthesis of proteins, sugars, and proline [15,16]. Notably, doping CDs with elements such as nitrogen and carbon further enhances their functional performance [17]. Nitrogen, a vital macronutrient involved in many physiological processes, not only improves the physicochemical properties of CDs but also contributes directly to plant growth and stress resilience [18]. These features make nitrogen-doped carbon dots (N-CDs) particularly attractive for mitigating environmental stress and improving crop resilience. However, until now, it remains unknown whether N-CDs are involved in tomato growth and pesticide detoxification processes.

Tomato (*Solanum lycopersicum* L.) is one of the most widely cultivated vegetable crops globally. To control fungal pathogens and maintain productivity, intensive fungicidal applications of chlorothalonil (CHT) are commonly employed. However, this fungicide often accumulates in leaves and fruits, inducing oxidative damage, disrupting photosynthesis, and impairing cellular metabolism in the plant. In this study, we synthesized and comprehensively characterized a series of N-CDs formulations (N-CDs1~N-CDs5) and evaluated their effects on CHT residue, photosynthetic efficiency, oxidative stress markers, antioxidant enzyme activities, and GSH metabolism in tomato. We aimed to elucidate the mechanisms by which N-CDs alleviate CHT-induced phytotoxicity and to assess their potential as effective, non-cytotoxic nanobiostimulants for CHT residue management in tomato.

## 2. Results

### 2.1. Synthesis of N-CDs with High Biological Safety

**Figure 1:**
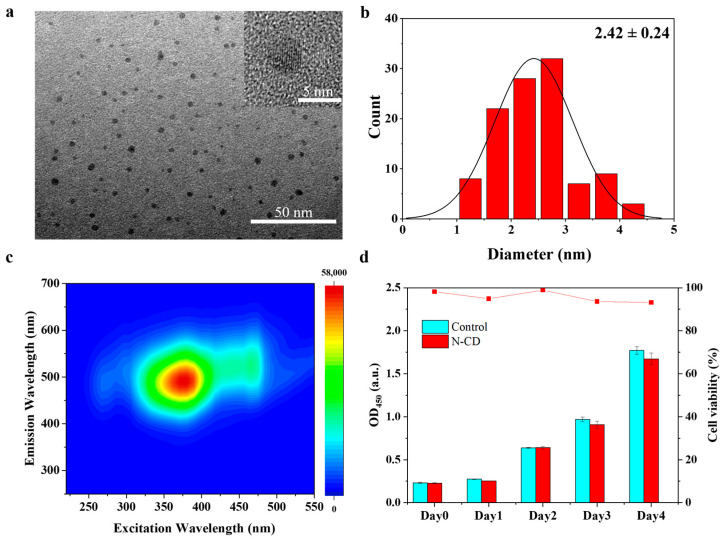
Verification of the morphological characteristics, fluorescence properties and safety of N-CDs. (**a**) Transmission electron microscopy (TEM) results. The scale bar represents 50 nm. (Inset: High-resolution TEM image with a scale bar representing 5 nm). (**b**) Size distribution of N-CDs. (**c**) Fluorescence 3D scanning spectra of N-CDs. (**d**) Cytotoxicity test.

N-doped citric acid precursor N-CDs were synthesized from citric acid and urea, with urea serving as the nitrogen source. The morphology of the fabricated materials was characterized by TEM (Figure 1a), revealing a uniform size and high monodispersity. The size distribution of the N-CDs ranged from 1 to 4.5 nm, with an average particle size of (2.42 ± 0.24) nm (Figure 1b). The fluorescence 3D scanning spectrum showed that the N-CDs in aqueous solution emitted intense blue fluorescence under excitation light ranging from 315 to 500 nm (Figure 1c). Furthermore, the biosafety of N-CDs was evaluated using human embryonic kidney 293T (HEK 293T) cells. The histogram in Figure 1d shows the absorbance at 450 nm (indicative of cell viability) for cultures treated with N-CDs and the blank control over four days. The corresponding line graph illustrates the relative survival rate of the N-CD-treated cells compared to the control. Four days later, the cell survival rate in the N-CD-treated group decreased to 93.14%; however, no significant differences were observed between the treated and control groups. These results indicate that the as-prepared N-CDs in the experiment possessed high biocompatibility and are promising for practical applications.

### 2.2. Analysis of Elemental Groups of N-CDs

**Figure 2:**
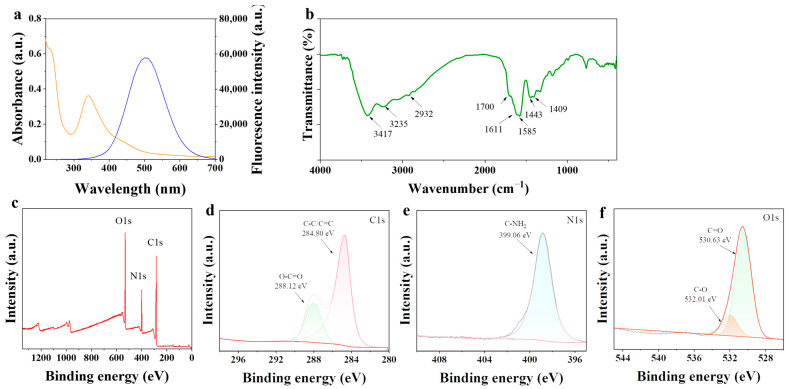
Characterization of elemental groups of N-CD. (**a**) UV/Vis absorption and fluorescence emission spectra of N-CDs (**b**) Fourier transform infrared spectroscopy (FT-IR) spectra. (**c**) X-ray photoelectron spectroscopy (XPS) spectrum of N-CDs. (**d**) The deconvolution graph of the C 1s spectrum of N-CDs. (**e**) The N 1s spectral deconvolution map of N-CDs. (**f**) The O 1S spectral deconvolution map of N-CDs.

The ultraviolet-visible (UV/Vis) spectrum of the prepared N-CDs aqueous solution was investigated (Figure 2a). The spectrum (orange line) of N-CDs exhibited strong absorption bands at 235 nm and 340 nm. The first absorption peak at 235 nm was attributed to the π-π* transition of the sp^2^ domain. The peak at 340 nm corresponded to n-π* transitions of C=O or C=N bonds. The optimal excitation wavelength was 378 nm. Upon excitation at this wavelength, the N-CDs emitted the most intense blue fluorescence at 488 nm (Figure 2a, blue line).

The surface functional groups of the N-CDs were characterized by Fourier transform infrared (FT-IR) spectroscopy. As illustrated in Figure 2b, the broad peak near 3200 cm^−1^ was associated with f N–H stretching vibration. The strong peak at 3417 cm^−1^ was assigned to the stretching vibration of the intermolecular hydrogen bond O–H. The absorption peak at 2932 cm^−1^ corresponded to C–H stretching vibration. The peak at 1443 cm^−1^ was the fingerprint region of C–H. The characteristic absorption peak at 1700 cm^−1^ was associated with the stretching vibration of C=O and C=C. The absorption peak at 1611 cm^−1^ was related to the bending vibration of the primary amine (N–H), and the peak near 1585 cm^−1^ was also assigned to N–H bending. The peak at 1409 cm^−1^ was caused by C–N stretching vibration. These peaks demonstrate that the surface of the N-CDs was rich in carboxyl, ester, hydroxyl, and carbonyl.

Furthermore, the X-ray photoelectron spectroscopy (XPS) survey spectrum indicated that the N-CDs were composed primarily of carbon, nitrogen, and oxygen with an atomic ratio of 62:15:23 (Figure 2c). The chemical states of these elements were further analyzed by using fine XPS spectra. As shown in Figure 2d–f, the C 1s spectrum (Figure 2d) could be deconvoluted into two peaks at 284.8 eV (C–C/C=C) and 288.12 eV (C=O/C=N). The N 1s spectrum (Figure 2e) was fitted with a single peak at 399.06 eV (N–H). The O1s spectrum (Figure 2f) was fitted with two peaks of C=O at 530.63 eV and C–O at 532.01 eV. Combined with FT-IR results, these XPS data confirmed that the N-CDs possessed abundant hydrophilic functional groups.

### 2.3. N-CDs Treatment Decrease CHT Residue in Tomato

**Figure 3:**
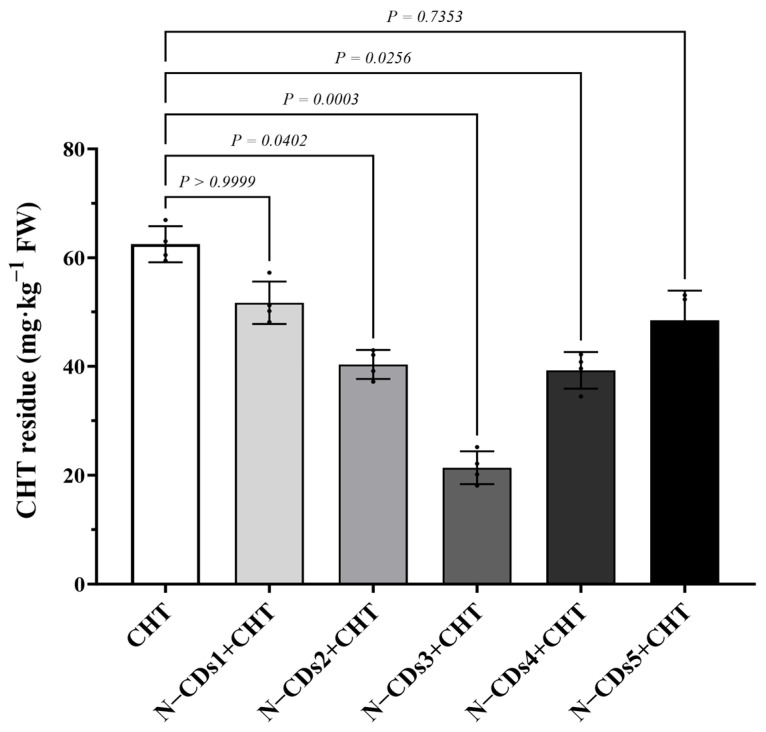
Effect of different concentrations of N-CD on chlorothalonil (CHT) residue in tomato. Two-week-old tomato plants were sprayed with N-CDs1 (50 mg·L^−1^), N-CDs2 (100 mg·L^−1^), N-CDs3 (150 mg·L^−1^), N-CDs4 (200 mg·L^−1^), and N-CDs5 (300 mg·L^−1^). Two days later, the plants were sprayed with either water (control) or 37.6 mM CHT. The leaf samples were collected seven days after CHT treatment for analyzing CHT residues. Each dot represented a biological repetition. Statistical significance was determined using one-way ANOVA followed by *Kruskal-Wallis* testing. Data are the mean ± SD from at least four biological replicates. There was a significant difference when *p* < 0.05, but no significant difference when *p* ≥ 0.05.

To analyze the effect of N-CDs on CHT residue in tomato leaves, different concentrations of N-CDs (N-CDs1~N-CDs5) were exogenously sprayed onto tomato leaves, followed by foliar application of CHT. Then, CHT residue in the tomato leaves was analyzed by high performance liquid chromatography (HPLC). Treatment with N-CDs1 to N-CDs5 significantly decreased CHT residue in tomato leaves compared to the control (Figure 3). Notably, the N-CDs3 treatment displayed the best effect, reducing CHT residues to 34% of the level in the control group (Figure 3). This result suggested that a concentration of 150 mg·L^−1^ N-CDs may be the most effective for decreasing CHT residue in tomato.

### 2.4. N-CDs Treatment Alleviated CHT-Induced Toxicity in Tomato

**Figure 4:**
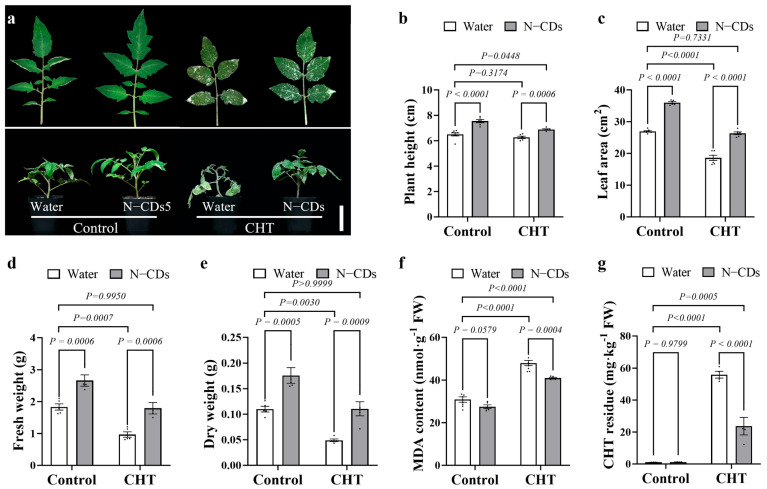
N-CDs treatment alleviated CHT-induced toxicity in tomato. (**a**) Phenotype of control and N-CDs sprayed tomato plants under control or CHT treatment. The scale bar represents 5 cm. Plant height (**b**), leaf area (**c**), dry weight (**d**), fresh weight (**e**), MDA concentration (**f**), and CHT residue (**g**) were measured in the indicated plants, respectively. Two-week-old tomato plants were sprayed with N-CDs (150 mg·L^−1^). Two days later, the plants were sprayed with either water (control) or 37.6 mM CHT. The plant samples were collected seven days after CHT treatment, for analysis of the related indicators. Each dot represented a biological repetition. Statistical significance was determined using two-way ANOVA followed by *L.S.D.* testing. Data are the mean ± SD from at least four biological replicates. There was a significant difference when *p* < 0.05, but no significant difference when *p* ≥ 0.05.

Thus, the 150 mg·L^−1^ N-CDs treatment was selected to analyze its effect on alleviating CHT-induced phytotoxicity in tomato plants. As shown in Figure 4a, N-CDs treatment enhanced tomato plant growth under both control and CHT conditions. Importantly, N-CDs-treated tomatoes under CHT stress exhibited growth similar to that of water-treated plants under control condition. In addition, plant height, leaf area, fresh weight, and dry weight were significantly increased by N-CDs treatment in both the control and CHT groups (Figure 4b–e). Interestingly, these growth indexes in CHT stressed plants treated with N-CDs recovered to levels comparable to those of the water treated control plants.

Then, malondialdehyde (MDA) content was measured to assess the extent of lipid peroxidation under CHT stress (Figure 4f). The accumulation of MDA was significantly reduced in N-CDs-treated tomatoes compared with water-treated tomatoes under CHT conditions. CHT residues were also significantly reduced in the N-CDs-treated tomatoes compared with the water-treated tomatoes (Figure 4f). These data indicated that N-CDs treatment could alleviate CHT-induced toxicity and decrease CHT residue accumulation in tomato plants.

### 2.5. N-CDs Treatment Alleviated CHT-Induced Depression in Photosynthesis in Tomato

**Figure 5:**
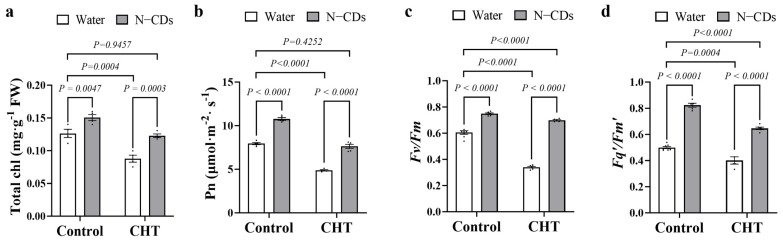
N-CDs treatment alleviated CHT-induced depression in photosynthesis in tomato. (**a**–**d**) represented total chl, Net photosynthetic rate (Pn), the maximum quantum yield of photosystem II (*Fv*/*Fm*), and the photochemical efficiency of photosystem II in the light (*Fv’*/*Fm’*), respectively. Two-week-old tomato plants were sprayed with N-CDs (150 mg·L^−1^). Two days later, the plants were sprayed with either water (control) or 37.6 mM CHT. The leaves were used for analyzing the related indicators at seven days after CHT treatment. Each dot represented a biological repetition. Statistical significance was determined using two-way ANOVA followed by *L.S.D.* testing. Data are the mean ± SD from at least four biological replicates. There was a significant difference when *p* < 0.05, but no significant difference when *p* ≥ 0.05.

Furthermore, photosynthetic capacity was also investigated (Figure 5). Under control conditions, total chlorophyll (chl) content, photosynthetic rate (Pn), the maximum quantum efficiency of PSII (*Fv*/*Fm*) level, and the efficiency of PSII photochemistry (*Fq’*/*Fm’*) were significantly increased in N-CDs-treated tomatoes. Under CHT conditions, photosynthetic capacity in all the tomato plants was significantly inhibited. However, the total chl content and Pn of N-CDs-treated tomatoes under CHT conditions recovered to those in the water-treated control plants (Figure 5a,b). In addition, N-CDs treatment under CHT conditions significantly increased the *Fv/Fm* and *Fq’/Fm’* levels compared to the water-treated control (Figure 5c,d). These results meant that N-CDs treatment alleviated CHT-induced depression in photosynthesis in tomato, potentially by promoting chl synthesis.

### 2.6. N-CDs Treatment Repressed ROS Accumulation and Activated the Antioxidant Enzyme Activity in Tomato Under CHT Condition

**Figure 6:**
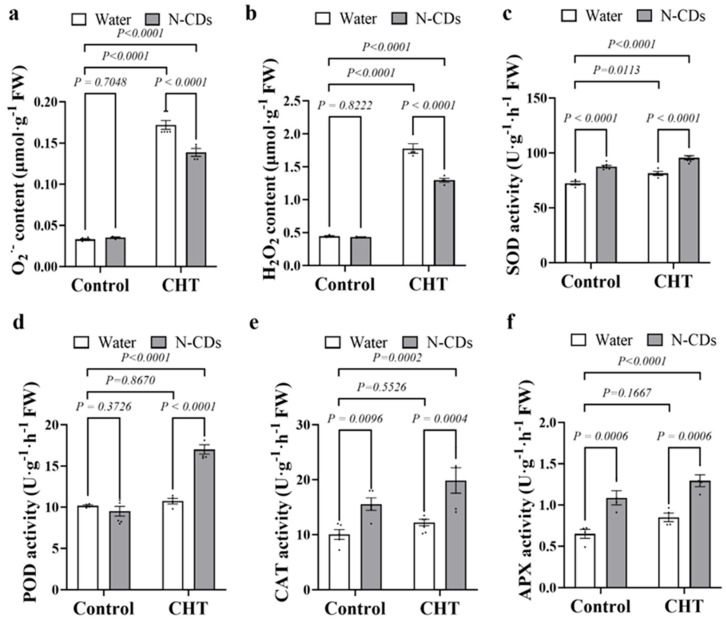
N-CDs treatment activated the antioxidant enzyme activity and repressed ROS accumulation in tomato. (**a**) H_2_O_2_ content, (**b**) O_2_^•−^ content, (**c**) superoxide dismutase (SOD) activity, (**d**) peroxidase (POD) activity, (**e**) catalase (CAT) activity and (**f**) ascorbate peroxidase (APX) activity were measured in the indicated plants, respectively. Each dot represents a biological repetition. Statistical significance was determined using two-way ANOVA followed by *L.S.D.* testing. Data are the mean ± SD from at least four biological replicates. There was a significant difference when *p* < 0.05, but no significant difference when *p* ≥ 0.05.

To evaluate whether N-CDs mediates tomato detoxification by affecting ROS accumulation, the levels of O_2_^•−^ and H_2_O_2_ were analyzed in tomato plants that were either water-treated or N-CDs-treated under both control and CHT conditions, respectively (Figure 6a,b). Under control conditions, the contents of O_2_^•−^ and H_2_O_2_ showed no significant difference between water-treated and N-CDs-treated tomatoes. Under CHT conditions, the contents of O_2_^•−^ and H_2_O_2_ were significantly enhanced in the leaves of all the tomato plants. However, the leaves of N-CDs-treated tomato plants accumulated significantly less O_2_^•−^ and H_2_O_2_ than water-treated tomatoes.

Then, the activities of antioxidant enzymes, including SOD, POD, CAT, and APX, were measured to analyze the N-CDs-mediated ROS scavenging ability under CHT conditions. As shown in Figure 6c–f, under control conditions, the activities of SOD, CAT, and APX significantly increased in N-CDs-treated tomatoes compared with water-treated tomatoes. Importantly, under CHT conditions, the activities of SOD, POD, CAT, and APX were further activated in N-CDs-treated tomatoes compared with those in water-treated tomatoes (Figure 6c–f).

### 2.7. N-CDs Treatment Enhance the Detoxification Potential of Tomato

**Figure 7:**
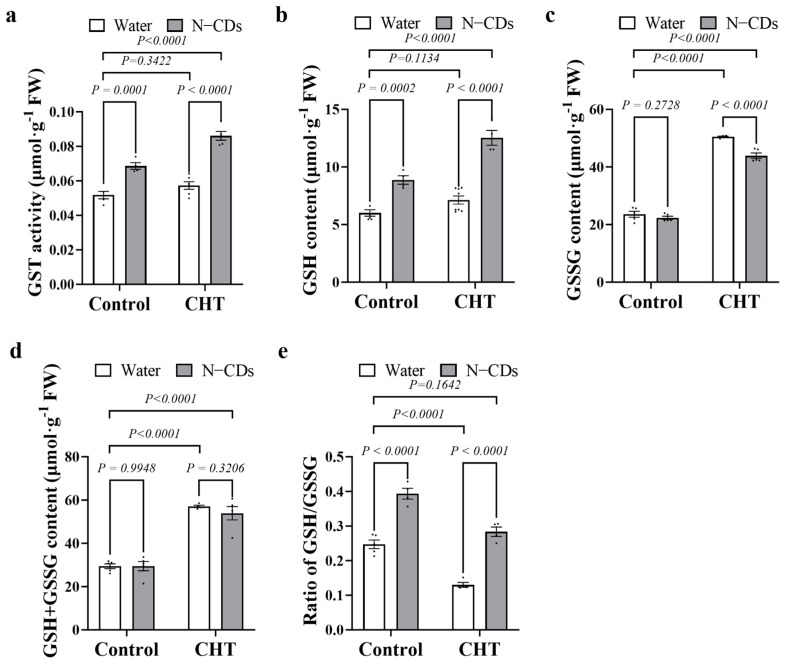
N-CDs treatments altered the synthesis and metabolism of glutathione in tomato. (**a**) Glutathione S-transferases (GST) activity, (**b**) glutathione (GSH) content, (**c**) oxidized glutathione (GSSG) content, (**d**) total glutathione content (GSH + GSSG), (**e**) ratio of GSH/GSSG was measured in the indicated plants, respectively. Each dot represented a biological repetition. Statistical significance was determined using two-way ANOVA followed by *L.S.D.* testing. Data are the mean ± SD from at least four biological replicates. There was a significant difference when *p* < 0.05, but no significant difference when *p* ≥ 0.05.

GSH and GST play crucial roles in the process of alleviating pesticide toxicity in plants. Therefore, the activity of GST, and the contents of GSH, GSSG, and GSH + GSSG, and the ratio of GSH/GSSG were analyzed, respectively. As shown in Figure 7c–e, CHT treatment significantly increased the content of GSSG and GSH + GSSG, but it drastically decreased the ratio of GSH/GSSG compared to the water-treated tomatoes. In contrast, under CHT conditions, N-CDs treatment significantly increased GST activity and GSH content by 50% and 75%, respectively, and reduced the content of GSSG by 14% compared to the water-treated tomatoes (Figure 7a–c). Notably, although N-CDs application did not affect the GSH content under control conditions, it increased the content of GST, leading to an increased ratio of GSH/GSSG (Figure 7a,b). Importantly, changes in GSH and GSSG resulted in a significant reduction in the ratio of GSH/GSSG under CHT conditions. However, N-CDs pretreatment significantly augmented the ratio of GSH/GSSG in N-CDs-treated tomatoes compared with the water-treated tomatoes under CHT conditions (Figure 7e).

## 3. Discussion

Pesticide residues accumulate in edible plant tissues and pose serious phytotoxic risks, including shoot apical atrophy, leaf necrosis, and impaired photosynthesis, ultimately reducing crop yield and threatening food safety [19]. Traditional approaches such as breeding for resistance or reducing application rates have only partially addressed this challenge. CDs offer high biocompatibility, water solubility, and environmental safety [20,21]. Previous studies have reported that the carbon dots could promote plant growth and improve plant stress tolerance by enhancing the photosynthetic capacity [17]. However, their ability to alleviate pesticide-induced phytotoxicity in plants had remained unexplored. Here, we synthesized N-CDs and found that the spraying N-CDs could dramatically reduce pesticide residues in tomato leaves (Figure 3). This result indicates that the alleviation of pesticide phytotoxicity by N-CDs may be attributed to their ability to enhance photosynthetic capacity. Generally, the smaller the particle size of carbon QDs, the more their fluorescence emission positions tend toward shorter wavelengths [22]. Our data confirm this trend (Figure 1). This ultra-small size was conducive to the efficient entry of N-CDs into plant cells to exert their potential biological functions. Previous studies have reported that citrazinic acid is a major contributor to the blue fluorescence of CDs derived from citric acid precursors [23]. In the presence of urea, green fluorescent molecular clusters such as 4-hydroxy-1*H*-pyrrole [3,4-*c*] pyridine-1,3,6(2*H*,5*H*)-trione (HPPT) can be generated during the preparation of CDs, with their abundance increasing as the proportion of urea rises [24]. In this work, the N-CDs synthesized from citric acid and urea emitted strong blue fluorescence (Figure 1), which may be attributed to differences in precursor ratios and synthesis conditions affecting the final product’s properties. Furthermore, based on UV/Vis spectrum, FT-IR, and XPS analyses (Figure 2), it was determined that the fluorescence of N-CDs was primarily due to citrazinic acid. Some carbon-based (like graphene oxide) and metal-based (like zinc oxide and potassium permanganate) nanomaterials exhibit a certain degree of cytotoxicity, with cell survival rates lower than 80% [25,26]. Chemical modifications (such as fetal bovine serum protein and l-cysteine, etc.) can reduce cytotoxicity [27,28]. Importantly, N-CDs exhibited high biological safety in cytotoxicity assays (Figure 1d), which might be attributed to the reliable safety of the precursor substance itself [29], showing potential for application in practical agricultural production.

Plants mount defensive responses to various pesticides, such as CHT, which are known to induce the production of ROS [19]. Excessive ROS accumulation can lead to oxidative stress and damage plant cells, thereby limiting the growth and development of plants [30]. Consistent with this, the pesticide treatment led to excessive ROS accumulation in tomatoes, reduced photosynthetic capacity, and stunted plant growth (Figure 4, Figure 5 and Figure 6). Importantly, the levels of ROS were significantly reduced in N-CDs-treated tomatoes (Figure 6a,b). Moreover, both photosynthetic performance and growth parameters recovered to normal levels in N-CDs-treated tomatoes (Figure 4 and Figure 5). However, oxidative damage at the cellular level may reduce membrane integrity. We observed that under pesticide treatment, the MDA concentrations increased significantly in all plants but were markedly reduced in N-CDs-treated tomatoes (Figure 4f). This result suggests that N-CDs-treated tomatoes had a batter membrane stability. For sustaining redox homeostasis, plants can boost antioxidant enzyme activities to mitigate oxidative damage and enhance their stress adaptability [31]. As the initial defense against excessive ROS, SOD transforms O_2_^•−^ into H_2_O_2_, which is then detoxified into water and oxygen by POD, CAT, and APX [32]. In this study, the activities of SOD, POD, CAT, and APX were more significantly activated in N-CDs-treated tomatoes than in water-treated tomatoes (Figure 6c–f). These results demonstrate that N-CDs treatment improved tomato tolerance to drought and salt stresses through decreasing ROS generation and maintaining a strong ROS-scavenging ability. Some studies have found that nanomaterials enhance photosynthesis [33] and improve salt tolerance [34] by activating the antioxidant enzyme system, which is consistent with our research results for plants under pesticide stress. These results indicate that the regulation of plant abiotic stress resistance by CDs may be inseparable from the antioxidant enzyme system. However, it remains unclear whether this impact was caused by the direct interaction of nanoparticles and antioxidant enzymes or was an indirect function of ROS-triggered signaling. In addition, previous studies have reported biostimulants capable of degrading CHT. For example, melatonin treatment reduced pesticide residues by 57% [6], while γ-aminobutyric acid (GABA) achieved a reduction of 45% [35]. Notably, in this study, N-CDs3 at a concentration of 150 mg·L^−1^ demonstrated the most significant efficacy, reducing pesticide residues by approximately 66% (Figure 3). This degradation performance was more effective compared to other biostimulants. The weakened decomposition effect at a concentration of 200–300 mg·L^−1^ may be due to the reduced spacing of N-CDs at high concentrations, resulting in a self-aggregation effect [36] and a decrease in the effective concentration of N-CDs entering the leaves. These results indicate that N-CDs treatment alleviated pesticide-induced phytotoxicity in tomato through activation of the antioxidant enzyme system, suppression of ROS generation, and stabilization of the plasma membrane at an appropriate level.

During pesticide detoxification in plants, GST plays a central role by catalyzing the conjugation of GSH to pesticide molecules [8]. We therefore profiled GST activity and the balance between reduced and oxidized GSH under CHT conditions. Under CHT conditions, the GSH/GSSG ratio decreased markedly, but N-CDs treatment restored this ratio to a healthier balance by enhancing antioxidant enzyme activity (Figure 6 and Figure 7). These results imply that N-CDs facilitated the conjugation of toxic compounds with GSH, thereby accelerating detoxification in tomato plants. GST is known to catalyze the conjugation of GSH to xenobiotics. Previous studies have reported that H_2_O_2_ signaling promotes both GSH synthesis and GST induction via melatonin treatment [19]. Notably, our findings align with the concept that redox shifts can upregulate detoxification enzymes. However, unlike melatonin, which elevates GSH levels even under control conditions, N-CDs treatment did not alter GSH pools in control plants but still boosted GST activity (Figure 7). This distinction may stem from mild, localized ROS signaling generated by the CDs’ surface, which selectively induces GST activity without broadly activating sulfur assimilation pathways. However, the roles played by Phase I (cytochrome P450s) and Phase III (ABC transporters) in the process of N-CDs alleviating pesticide toxicity still require further study.

**Figure 8:**
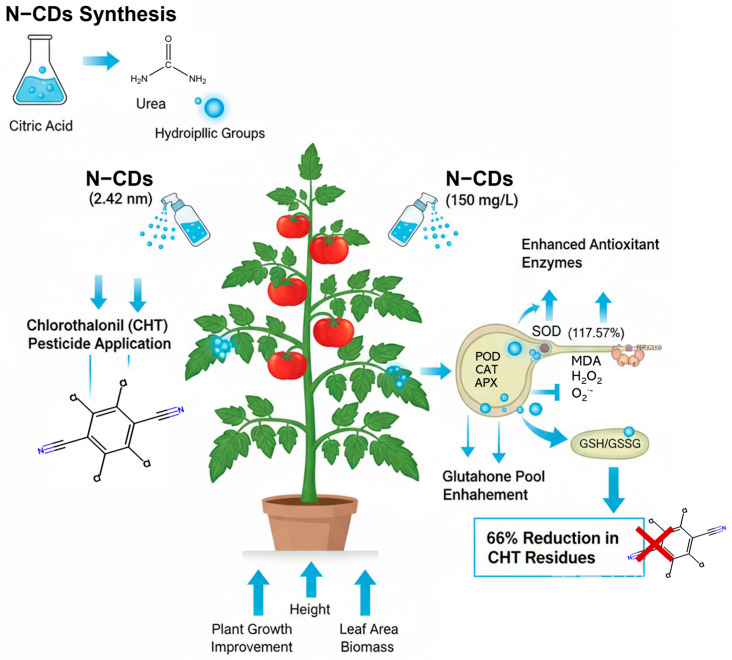
The mechanistic model of N-CDs in alleviating CHT toxicity in tomato.

Collectively, these results supported the conclusion that N-CDs mediated pesticide detoxification in tomato primarily through modulating redox homeostasis (Figure 8). In addition, N-CDs represent a novel agent of biostimulants that can fine-tune plant antioxidant defenses with minimal disruption under normal conditions. However, due to the potential species-specific toxicity of nanomaterials, future studies on plant seed and root will be needed to verify the plant-level safety of N-CDs. In this study, the growth-promoting and CHT-detoxifying effects of N-CDs were confirmed using the sequenced tomato cultivar ‘Heinz 1706′. Whether these findings are applicable to other varieties and pesticides requires further validation.

## 4. Materials and Methods

### 4.1. The Synthesis, Characterization, and Cytotoxicity Test of N-CDs

The preparation of N-CDs followed our previous method with minor modifications [37]. Briefly, 1.025 g citric acid and 0.379 g urea underwent hydrothermal reaction at 160 °C for 6 h. The resulting product was dialyzed (3.5 kDa) for 48 h and stored at 4 °C in the dark. The N-CDs were characterization using the following techniques: TEM (JEM-F200, JEOL, Tokyo, Japan) for morphology and size; UV/Vis spectroscopy (Shimadzu, UV-2600i) and fluorescence spectroscopy (LF-1701009, Thermo Fisher Scientific, Waltham, MA, USA) for optical properties; FT-IR (Thermo Fisher Scientific, NICOLET iS20, Waltham, MA, USA) and XPS (K-Alpha, Thermo Scientific, Waltham, MA, USA) for identifying surface functional groups and elemental composition. Cytotoxicity was evaluated according to our previous method [38]. HEK 293T cells were treated with N-CDs at a concentration of 10 mg·mL^−1^, and cell viability was assessed using the CCK-8 assay at 0, 24, 48, 72, and 96 h. The measurements of cytotoxicity were performed in triplicate and expressed relative to untreated controls.

### 4.2. Plant Materials and Pesticide Treatment

Tomato cultivar ‘Heinz 1706′ seeds were germinated and grown in a growth chamber. The seeds were surface-sterilized by immersion in 75% ethanol for 1 min, followed by treatment with 8% NaClO solution for 15 min. Then, the seeds were rinsed five times with sterile water and sown on plates containing Morishige and Skoog (MS) medium supplemented with sucrose. Dry seeds were directly sown at the varying depth in a soil mixture containing 40% organic matter in pots of 8 × 8 × 8.5 cm. All seedlings were grown in a growth chamber at 28 °C with 16 h light and 22 °C with 8 h light, as previously described [39].

For pesticide treatment, two-week-old tomato plants were sprayed with different concentrations of N-CDs: N-CDs1 (50 mg·L^−1^), N-CDs2 (100 mg·L^−1^), N-CDs3 (150 mg·L^−1^), N-CDs4 (200 mg·L^−1^), and N-CDs5 (300 mg·L^−1^). Two days later, the plants were treated with either water (control) or 37.6 mM CHT. Leaf samples were collected 7 days after CHT treatment, flash-frozen in liquid nitrogen, ground to a fine powder, and stored at −80 °C until analysis. All measurements were performed in three independent experiments with at least four biological replicates.

### 4.3. Growth Related Parameters Analysis

Plants were photographed at the indicated time points. Plant height and the area of the fifth leaf were measured at the indicated time points using Image J software (ij154-win-java8). The fresh weight (FW) of shoot was recorded immediately. For dry weight (DW) determination, samples were first heated at 105 °C for 20 min and then dried at 75 °C until constant weight was achieved [30]. All measurements were performed in three independent experiments with at least four biological replicates.

### 4.4. Photosynthetic Related Parameters Analysis

Total chlorophyll content was determined according to the previously described method [40]. Briefly, total chlorophyll was extracted from 0.05 g of tomato leaf tissue using 80% acetone. The absorbance of the extract was measured using a multi-mode microplate reader (Synergy H4 Hybrid) at OD_470_, OD_647_, and OD_663_. Total chlorophyll content was calculated using the following formula:

(1) $$chla\left(mg/L\right)=12.21\times {OD}_{663}-2.81\times {OD}_{647}$$

(2) $$chlb\left(mg/L\right)=20.13\times {OD}_{647}-5.03\times {OD}_{663}$$

(3) $$Total\;chl\left(mg/L\right)=\frac{1000\times {OD}_{647}-3.27\times chla-104\times chlb}{229}$$

Pn was measured with a Li-6400 portable gas-exchange system [41]. The *Fv*/*Fm* and the *Fq’*/*Fm’* in tomato leaves were assessed using an FMS-2 Pulse Modulated Fluorometer (Hansha Scientific Instruments, Beijing, China) [5]. All measurements were performed three independent experiments with at least four biological replicates.

### 4.5. ROS Contents and Antioxidant Enzyme Activities Analysis

The contents of O_2_^•−^ and H_2_O_2_ and the activity of SOD, POD, CAT, and APX in tomato leaves were measured based on previously described methods [5]. Lipid peroxidation was evaluated by measuring MDA using the TBARS method [42]. In detail, the O_2_^•−^ content was extracted from 0.05 g of tomato leaf tissue using a commercial assay kit (BC1295, Beijing Solarbio Science & Technology Co., Ltd., Beijing, China) by measuring absorbance at 530 nm. The concentration was calculated according to the following equation (C_standard_ = 0.03125 μmol·mL^−1^, V = 0.1 mL, and W = 0.05 g):

(4) $$Content=2\times C_{standard}\frac{A_{sample}-A_{blank}}{A_{standard}-A_{blank}}\times \frac{V}{W}$$

The H_2_O_2_ content was extracted from 0.05 g of tomato leaf tissue using a commercial assay kit (BC3595, Beijing Solarbio Science & Technology Co., Ltd.) by detecting absorbance at 415 nm. The content was also calculated by Equation (4) (C_standard_ = 2 μmol·mL^−1^, V = 0.1 mL, and W = 0.05 g):

In addition, 0.1 g of tomato leaf samples were homogenized in 0.05 mol·L^−1^ phosphate buffer (pH 7.8) and centrifuged to obtain the crude enzyme extract. The supernatant was subsequently used for the analysis of enzyme activities and MDA content.

SOD activity was assayed by adding 25 μL of crude enzyme extract to 18 μL of 0.6 mg/mL nitrobluetetrazolium (NBT), followed by absorbance measurement at 560 nm. SOD activity was calculated as follows:

(5) $$SOD\;content=\left(A_{light}-A+A_{dark}\right)\times \frac{V}{{0.5\times A}_{light}\times W\times V_{t}},$$

where A_light_ = absorbance of the light control, A_dark_ = absorbance of the dark control, A = absorbance of the sample, W = 0.1 g, V = 0.6 mL, and Vₜ = 0.025 mL.

POD activity was measured by mixing 20 μL of crude enzyme extract with 28 μL of 100% guaiacol. Absorbance at 470 nm was recorded at 1-min intervals for a total of 16 readings. POD activity was calculated using Formula (6). CAT activity was determined by adding 5 μL of crude enzyme extract to 50 μL of 0.1 mol/L hydrogen peroxide. The decrease in absorbance was monitored at 240 nm. CAT activity was also calculated according to Formula (6). APX activity was assessed by incubating 5 μL of crude enzyme extract with 100 μL of 0.88 mg/mL ascorbate hydrogen peroxide. Absorbance was measured at 560 nm. APX activity was calculated as follows:

(6) $$Total\;POD\;activity=\frac{∆A\times V}{a\times W\times t},$$

where ΔA = [|Aₜ_1_ − Aₜ_0_| + |Aₜ_2_ − Aₜ_1_| + … + |Aₜ_15_ − Aₜ_14_|]/15, V = 0.6 mL, a = 0.01 mL, W = 0.1 g, and t = 1 min.

The MDA content was determined by mixing 300 μL of crude enzyme extract with 0.67% thiobarbituric acid (TBA). Absorbance was measured at 450, 532, and 600 nm. The MDA content was calculated using the following equation:

(7) $$MDA\;content=C\times \frac{V}{W},$$

where C = 6.45 × (A_532_ − A_600_) − 0.56 × A_450_, V = 3 mL, and W = 0.1 g.

All spectrophotometric measurements were performed using a Synergy H4 Hybrid Reader (BioTek Instruments, Winooski, VT, USA). All measurements were performed in three independent experiments with at least four biological replicates.

### 4.6. Analysis of CHT Content by HPLC

The CHT content in tomato leaves was quantified by HPLC according to a previously reported method with minor modifications [43]. In detail, 0.2 g of sample was extracted with 2.0 mL of acetonitrile and 0.2 g of NaCl, vortexed thoroughly, and incubated at room temperature for 30 min. The supernatant was collected and evaporated to near dryness under a gentle stream of nitrogen at 60 °C. The residue was reconstituted in 2.0 mL of n-hexane. The extract was purified using an alumina-N solid-phase extraction column cartridge preconditioned sequentially with n−hexane/acetone and n−hexane. After loading the sample, the cartridge was rinsed, and the eluate was collected. The eluate was evaporated at 50 °C and reconstituted in 2.0 mL mobile phase. The final solution was filtered through a 0.22 μm membrane filter prior to HPLC injection. A stock solution of CHT (1.0 mg·mL^−1^) was prepared in acetonitrile and stored at −20 °C. Working standards of 0.05, 0.1, 0.5, 1, 2, 5, 10, 50, 100, and 200 mg·L^−1^ CHT was prepared by gradient dilution of the stock solution with the mobile phase before analysis. HPLC analysis was performed under the following conditions: column: ZORBAX Eclipse XDB-C18 (250 mm × 4.6 mm, 5 μm); mobile phase: acetonitrile/water (55:45, *v*/*v*); flow rate: 0.6 mL·min^−1^; detection: 232 nm; injection volume: 10 μL; column temperature: 30 °C. All measurements were performed in three independent experiments with at least four biological replicates.

### 4.7. Data Analysis

Data were compiled in Microsoft Excel. Means and standard deviations were calculated from biolobical replicates using Graphpad Prism software. Statistical significance was determined using one-way ANOVA followed by Kruskal-Wallis testing or two-way ANOVA followed by *L.S.D.* testing, also conducted in Graphpad Prism software (version 10.6.1). All data are presented as mean ± SD from at least four biological replicates. *, **, ***, and **** indicate significant differences at *p* < 0.05, *p* < 0.01, *p* < 0.001, and *p* < 0.0001, respectively.

## 5. Conclusions

In conclusion, this study demonstrates that N-CDs are non-cytotoxic and effective nanobiostimulants for enhancing plant growth and mitigating pesticide-induced stress in tomato. The hydrothermally synthesized N-CDs (∼2.4 nm), rich in hydrophilic groups and exhibiting strong blue fluorescence, dramatically reduced CHT residues by up to 66% at 150 mg·L^−1^. Under CHT conditions, N-CD treatment restored plant growth parameters including height, leaf area, and biomass to levels comparable with the water-treated tomato. These improvements coincided with reduced levels of oxidative stress markers (MDA, O_2_^•−^, H_2_O_2_) and enhanced the activities of antioxidant enzymes (SOD, POD, CAT, APX), alongside an expansion of the GSH pool. Collectively, our findings indicated that N-CDs strengthen the antioxidant defense system to promote detoxification in tomato.

## Data Availability

Data is contained within the article.
